# SPHINX-Based Combination Therapy as a Potential Novel Treatment Strategy for Acute Myeloid Leukaemia

**DOI:** 10.3389/bjbs.2023.11041

**Published:** 2023-02-21

**Authors:** Chigeru Wodi, Tareg Belali, Ruth Morse, Sean Porazinski, Michael Ladomery

**Affiliations:** ^1^ Centre for Research in Bioscience, Faculty of Health and Applied Sciences, University of the West of England, Bristol, United Kingdom; ^2^ Garvan Institute of Medical Research, Darlinghurst, NSW, Australia; ^3^ St Vincent’s Clinical School, Faculty of Medicine, University of New South Wales, Sydney, NSW, Australia

**Keywords:** acute myeloid leukemia, splice factor kinases, SRPK1, alternative splicing, SPHINX

## Abstract

**Introduction:** Dysregulated alternative splicing is a prominent feature of cancer. The inhibition and knockdown of the SR splice factor kinase SRPK1 reduces tumour growth *in vivo*. As a result several SPRK1 inhibitors are in development including SPHINX, a 3-(trifluoromethyl)anilide scaffold. The objective of this study was to treat two leukaemic cell lines with SPHINX in combination with the established cancer drugs azacitidine and imatinib.

**Materials and Methods:** We selected two representative cell lines; Kasumi-1, acute myeloid leukaemia, and K562, BCR-ABL positive chronic myeloid leukaemia. Cells were treated with SPHINX concentrations up to 10μM, and in combination with azacitidine (up to 1.5 μg/ml, Kasumi-1 cells) and imatinib (up to 20 μg/ml, K562 cells). Cell viability was determined by counting the proportion of live cells and those undergoing apoptosis through the detection of activated caspase 3/7. SRPK1 was knocked down with siRNA to confirm SPHINX results.

**Results:** The effects of SPHINX were first confirmed by observing reduced levels of phosphorylated SR proteins. SPHINX significantly reduced cell viability and increased apoptosis in Kasumi-1 cells, but less prominently in K562 cells. Knockdown of SRPK1 by RNA interference similarly reduced cell viability. Combining SPHINX with azacitidine augmented the effect of azacitidine in Kasumi-1 cells. In conclusion, SPHINX reduces cell viability and increases apoptosis in the acute myeloid leukaemia cell line Kasumi-1, but less convincingly in the chronic myeloid leukaemia cell line K562.

**Conclusion:** We suggest that specific types of leukaemia may present an opportunity for the development of SRPK1-targeted therapies to be used in combination with established chemotherapeutic drugs.

## Introduction

Soon after the discovery of pre-mRNA splicing in the late 1970s it became apparent that pre-mRNAs are alternatively spliced so that a multi-exon gene can produce multiple transcripts through exon skipping, intron retention, and the use of alternative 5′ and 3′ splice sites. Over the years it became clear that alternative splicing is a widespread process in eukaryotic cells and that it accounts, to a very large extent, for the complexity of the proteome (1, 2). The vast majority of human multi-exon genes are alternatively spliced. Population-scale transcriptomic analysis reveals the presence of numerous genetic variants that affect splicing that influence phenotype including disease susceptibility (3). Splice isoforms can exhibit distinct biological properties (pro- or anti-apoptotic; pro- or anti-angiogenic, etc.), and it is now evident that the dysregulation of alternative splicing is implicated in all hallmarks of cancer (4). This presents opportunities for the development of novel cancer therapies. Oncogenic splice isoforms can be targeted directly; alternatively, regulators of alternative splicing can also be targeted.

Alternative splicing is principally regulated by splice factors that bind to specific sequences in pre-mRNA modifying the choice of specific splice sites. One important family of splice factors are the SR proteins. They generally consist of one or more RNA Recognition Motifs (RRMs) and a serine-arginine (SR) rich domain, the latter involved in protein-protein interactions. One of the most widely studied SR protein splice factors is SRSF1, a splice factor with a well-established involvement in cancer (5). The activity of splice factors is in turn, regulated by the activity of splice factor protein kinases. SRSF1 is phosphorylated by the SRPKs (SR protein kinases; SRPK1 and SRPK2 in humans) and CLKs (CDC2-like protein kinases, CLK1-4 in humans (6)). The SRPKs phosphorylate SRSF1 at multiple serines in the SR domain (7). Phosphorylation of SRSF1 by SRPKs in the cytoplasm is required for the accumulation of SR proteins in the nucleus, whereas its phosphorylation by CLKs regulates their association with nuclear speckles and their biochemical activity (8).

The broad developmental and physiological roles of splice factor kinases are not yet fully understood. In the nematode *Caenorhabditis elegans*, the SRPK splice factor kinase SPK-1 is essential for embryogenesis and germline development (8) and inhibits programmed cell death by modifying the alternative splicing of *ced-4*, the *C. elegans* orthologue of human *Apaf-1* (9). The substrates of SRPKs may not be limited to SR proteins, and therefore their functions extend beyond the regulation of alternative splicing. To illustrate their functional complexity, in fertilised mammalian oocytes, SRPK1 catalyses the site-specific phosphorylation of protamines, helping trigger the protamine to histone exchange required for paternal genome reprogramming (10).

Given their involvement in modulating splice factor activity, and in other processes, it is not surprising that splice factor kinase expression is dysregulated in cancer (11), even affecting therapeutic responses to chemotherapy and radiotherapy (12). The acetylation of SRPK1 by the histone acetyltransferase Tip60 alters the activity of SRPK1 and modulates alternative splicing. In cisplatin-resistant breast cancer cells, reduced acetylation of SRPK1 by Tip60 increases its activity, favouring the expression of anti-apoptotic splice variants (13). In breast cancer, elevated SRPK1 activity reduces apoptosis through RBM4-regulated splicing events (14). SRPK1’s role in cancer is not limited to the regulation of apoptosis. A migration screen based on a phagokinetic track assay identified SRPK1 as a determinant factor in breast cancer metastasis (15); and a separate study demonstrates that the LIM domain kinase 2 (LIMK2) promotes breast cancer metastasis through SRPK1 activation (16). SRPK1 is implicated in other cancer types, including colorectal cancer and leukaemia (17). SRPK1 is a poor prognostic indicator in colorectal cancer (18) in which it modulates SRSF1-mediated *MKNK2* alternative splicing (19) and is required for the expression of the cancer-associated splice variant RAC1B (20). Of significant recent interest is the work of Tzelepis and colleagues. A CRISPR-Cas9 platform was used to screen for genetic vulnerabilities in acute myeloid leukaemia (AML) and identified SRPK1 as a potential therapeutic target (21). The authors demonstrated that both genetic or pharmacological inhibition of SRPK1 prolonged the survival of murine AML models by altering the splicing of several leukemogenesis-associated genes including *MYB*, *MED24* and *BRD4* (22). Furthermore, SRPK1 has been implicated in other leukaemias, including chronic myeloid leukaemia (CML). Salesse et al. demonstrated that numerous pre-mRNA splicing proteins are overexpressed in patient-derived cell line models of CML, including SRPK1, suggesting that aberrant pre-mRNA splicing may contribute to CML pathogenesis (23). Together, these studies suggest the therapeutic potential of targeting SRPK1 in a variety of cancers, including blood-borne cancers.

To further underline the oncogenic activity of SRPK1, we have previously identified SRPK1 as a key regulator of *VEGFA* alternative splicing (24). SRPK1 promotes the expression of pro-angiogenic VEGFA through the phosphorylation of SRSF1. This causes nuclear accumulation of SRSF1 which then promotes the use of a proximal 3′ splice site in exon 8, favouring the expression of the pro-angiogenic VEGFA splice isoform. Both the knockdown and pharmacological inhibition of SRPK1 shifts the ratio of splice isoforms in favour of the anti-angiogenic isoform of VEGFA165b (24), and we have proposed that targeting SRPK1 could be a viable avenue in prostate cancer (25).

There is considerable interest in developing novel and selective SRPK1 inhibitors. To this end Gammons and colleagues identified a 3-(trifluoromethyl)anilide scaffold named SPHINX that exhibits an IC_50_ of 880 nM for SRPK1 (26). SPHINX has been tested *in vivo* in the context of age-related macular degeneration (AMD), where it convincingly reduces choroidal neovascularization in rodents by promoting the expression of the anti-angiogenic VEGFA splice isoform (27). SPHINX also has potent effects on the growth and spread of orthotopic xenografts of human PC3 prostate cancer cells (25). The aim of the present research was to test the effect of SPHINX on two well-studied leukaemic cell line models (Kasumi-1 and K562) both alone, and in combination with established chemotherapeutic agents, azacitidine and imatinib.

## Materials and Methods

### Cell Culture and SPHINX Treatments

K562 and Kasumi-1 cell lines were purchased from the European Collection of Authenticated Cell Cultures (ECACC) and cultured using RPMI-1640 culture medium with L-glutamine (Sigma Aldrich, United Kingdom), further supplemented with 10% foetal bovine serum (FBS; Sigma Aldrich) for K562 cells and 20% FBS for Kasumi-1 cells. Cells were used between passages 6–19, seeded at densities of 5 × 10^5^–1 × 10^6^ in T25 flasks and were sub-cultured every 48 h. Cell lines were incubated in 5% CO_2_ at 37°C.

K562 and Kasumi-1 cells were treated with SRPK1 specific small molecule inhibitors 5-methyl-N-(2-(morpholin-4-yl)-5-(tri-fluoromethyl)phenyl) furan-2-carboxamide, commonly known as SR Protein Inhibitor X (SPHINX) which was purchased from Enamine (Kiev, Ukraine). SPHINX was dissolved in DMSO (Sigma Aldrich) at a stock concentration of 25 mM. Cells (1 × 10^6^/ml) were seeded in a T25 flask for each treatment which was performed in duplicate. Cells were incubated with 10 nM-10 μM SPHINX for up to 72 h.

### Cell Viability and Apoptosis Measurements

Cell counts and viability were determined using either trypan-blue staining and manual counting or using the Luna FL automated cell counter (Logos Biosystems, France). Percentage cell viability was calculated by dividing live cells over the total cell count.

Apoptosis was measured using the CellEvent Caspase-3/7 green detection reagent (ThermoFisher Scientific, United Kingdom) according to manufacturer’s instructions. Pelleted cells (20,000) were suspended in reagent and incubated for 45 min at room temperature. Counterstaining was performed with Hoechst for 1 min after which cells were transferred into a cytofunnel (ThermoFisher Scientific) and spun onto a microscope slide using the Cytospin 4 (ThermoFisher Scientific) at 20,000 g for 8 min. Slides were air-dried and mounted using Mowiol aqueous mounting media. Images were taken with a Nikon Eclipse 80i fluorescent microscope at ×40 magnification. For each treatment, green fluorescent cells were considered positive for activated caspase-3/7. For each slide, the total number of caspase positive cells in ten representative fields of view were recorded and calculated as a percentage of the total cells (positive and negative).

### SiRNA-Mediated SRPK1 Knockdown

For siRNA-mediated knockdowns, K562 and Kasumi-1 cells were cultured to 80% confluence. Cells were harvested and spun down to remove growth media. For each line, 5 × 10^5^ cells were pelleted and re-suspended in 800 µl of OptiMEM media (Gibco, United Kingdom) and transferred into 6-well plates and incubated in 5% CO_2_ at 37°C.

The SRPK1 siRNA (Eurofins, Genomics, United Kingdom) sequence was 5′-UUA​AUG​ACU​UCA​AUC​ACU​CCA​UUG​C-3′ and the scrambled siRNA control was 5′GCA​GCA​GCA​GCA​GCG​GGA​CTT-3′. Lipofectamine/OptiMEM cocktail (100 μl) (Thermo Fisher Scientific, United Kingdom) was added to 40 μl SRPK1 siRNA (2.5 mM) and incubated at room temperature for 20 min. Following this, siRNA/Lipofectamine/OptiMEM mixture was added to each well at a final concentration of 100 nM and incubated for 4 h, after which 1 ml of culture media was added to each well and cells were incubated for a further 48 h from the time of transfection.

### Cell Lysates and Western Blotting

Cell lysates were prepared using RIPA buffer (10 mM Tris-Cl (pH 8.0), 1 mM EDTA, 1% (v/v) Triton X-100, 0.1% (w/v) sodium deoxycholate, 0.1% (w/v) SDS and 140 mM NaCl) supplemented with protease inhibitor tablets (ThermoFisher Scientific). Equal protein samples (20 µg protein), were separated on 10% (v/v) SDS polyacrylamide gels and transferred to PVDF membranes (Sigma Aldrich) which were blocked in 2% (w/v) skimmed milk and probed overnight at 4°C with primary antibodies: anti-SRPK1 (EE-13, Santa Cruz Biotechnology; 1:1000) or anti-pan-SR-1H4 antibody (sc-13509, Santa Cruz Biotechnology; 1:500), anti-β-actin (ab8226, Abcam UK; 1:5000). Membranes were incubated in HRP-linked anti-rabbit or anti-mouse IgG secondary antibody (Cell Signalling; 1:1500) for 1 h at room temperature. Membranes were incubated in Luminata Forte Western HRP substrate (Millipore) for chemiluminescent detection prior to image acquisition which was performed using the LI-COR Odyssey FC imaging system (LI-COR, USA). Images acquired were exported to Image Studio Lite (LI-COR, USA) software for quantification. Experiments were performed in triplicate.

### Molecular Characterisation of Kasumi-1 and K562 Cell Lines

Mutation status and protein expression data were downloaded for all AML and CML cell lines available within the Cell Model Passports project, including Kasumi-1 and K562 cells (https://cellmodelpassports.sanger.ac.uk/) (28). Protein expression Z-scores for AML and CML cell lines were plotted as waterfall plots using GraphPad Prism (V9.0.0, GraphPad, United States).

### Statistical Analysis

Statistical analyses using ANOVAs or unpaired two-tailed t-tests were performed using GraphPad Prism. Significance levels are indicated by asterisks where *p < 0.05, **p < 0.01, ***p < 0.001 and ****p < 0.0001. Data are reported as means and error bars show standard error of the means.

## Results

### Effect of SPHINX on SR Protein Phosphorylation

Two leukaemic cell line models, Kasumi-1 and K562, were selected to investigate the effects of SPHINX. The Kasumi-1 cell line was derived from the peripheral blood of a patient with acute myeloblastic leukaemia (AML); K562 cells were derived from the pleural effusion of a patient with chronic myelogenous leukaemia (CML) and are BCR-ABL positive. The mutation status of SRPKs and SRSF splice factors in Kasumi-1 and K562 cell lines was initially examined using a publicly available dataset (https://cellmodelpassports.sanger.ac.uk/). This revealed no mutations in SRPK1 or SRPK2 in either cell line but demonstrated copy number changes for SRSF2 in Kasumi-1 and SRSF3 in K562. Furthermore, for SRSF12 (in Kasumi-1), these analyses identified a missense mutation (c.536G>A) which causes a replacement of arginine with glutamine at codon 179 at the protein level (p.R179Q; Supplementary Figure S1A). Additionally, the expression of SRPK1 and SRPK2 at the protein level in Kasumi-1 and K562 cells was examined and compared with other AML and CML cell lines available in the Cell Model Passports project (28). This identified that Kasumi-1 cells have comparatively lower levels of SRPK1 and SRPK2 versus K562 cells (Supplementary Figure S1B). When comparing SRPK1 and SRPK2 levels across a panel of AML cell lines, Kasumi-1 had the lowest SRPK1 expression and intermediate levels of SRPK2 comparatively (Supplementary Figure S1C). Across the CML cell line panel, K562 had comparatively high SRPK1 and SRPK2 levels (Supplementary Figure S1D), suggesting that Kasumi-1 and K562 might exhibit differential responses to SRPK1 inhibition by SPHINX.

**FIGURE 1:**
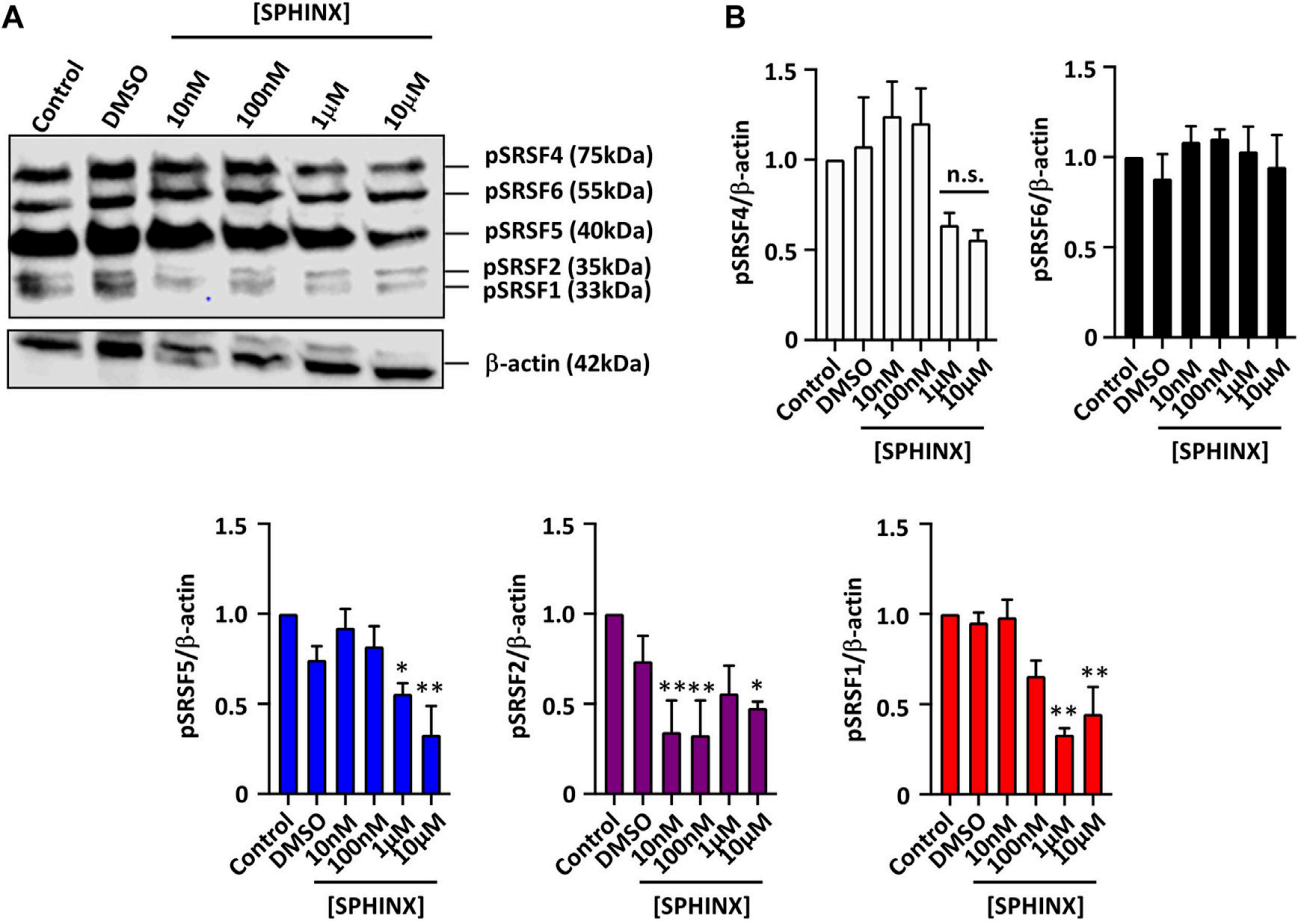
Reduction in phospho-SR protein levels in Kasumi-1 cells treated with SPHINX for 24 h. **(A)** Representative western blot image showing levels of SR protein phosphorylation, and time-matched β-actin. **(B)** Quantification of western blot image normalized with control showing a significant decrease in phosphorylated protein of SRSF5, SRSF2 and SRSF1 at higher concentration of SPHINX (*p < 0.05; **p < 0.01). One-way ANOVA (*n* = 3).

In order to assess the efficacy of SRPK1 inhibition by SPHINX on substrate phosphorylation, we previously showed that the treatment of PC3 prostate cancer cells with SPHINX led to reduced levels of phosphorylated SR proteins coinciding with increased expression of anti-angiogenic VEGFA (25, 29). SPHINX had the same effect on the leukaemic cell line Kasumi-1 using the mouse monoclonal antibody 1H4 which was specific to phosphorylated SR proteins (Figure 1A). Levels of phosphorylated SRSF1, SRSF2, and SRSF5 in Kasumi-1 cells were significantly reduced when cells were exposed to 1–10 μM of SPHINX for 24 h (Figure 1B). Conversely, in K562 cells, no change in pSRSF2, pSRSF4 and pSRSF5 protein levels was observed following SPHINX treatment for 24 h (Supplementary Figure S2).

### Effect of SPHINX on Cell Viability and Apoptosis

**FIGURE 2:**
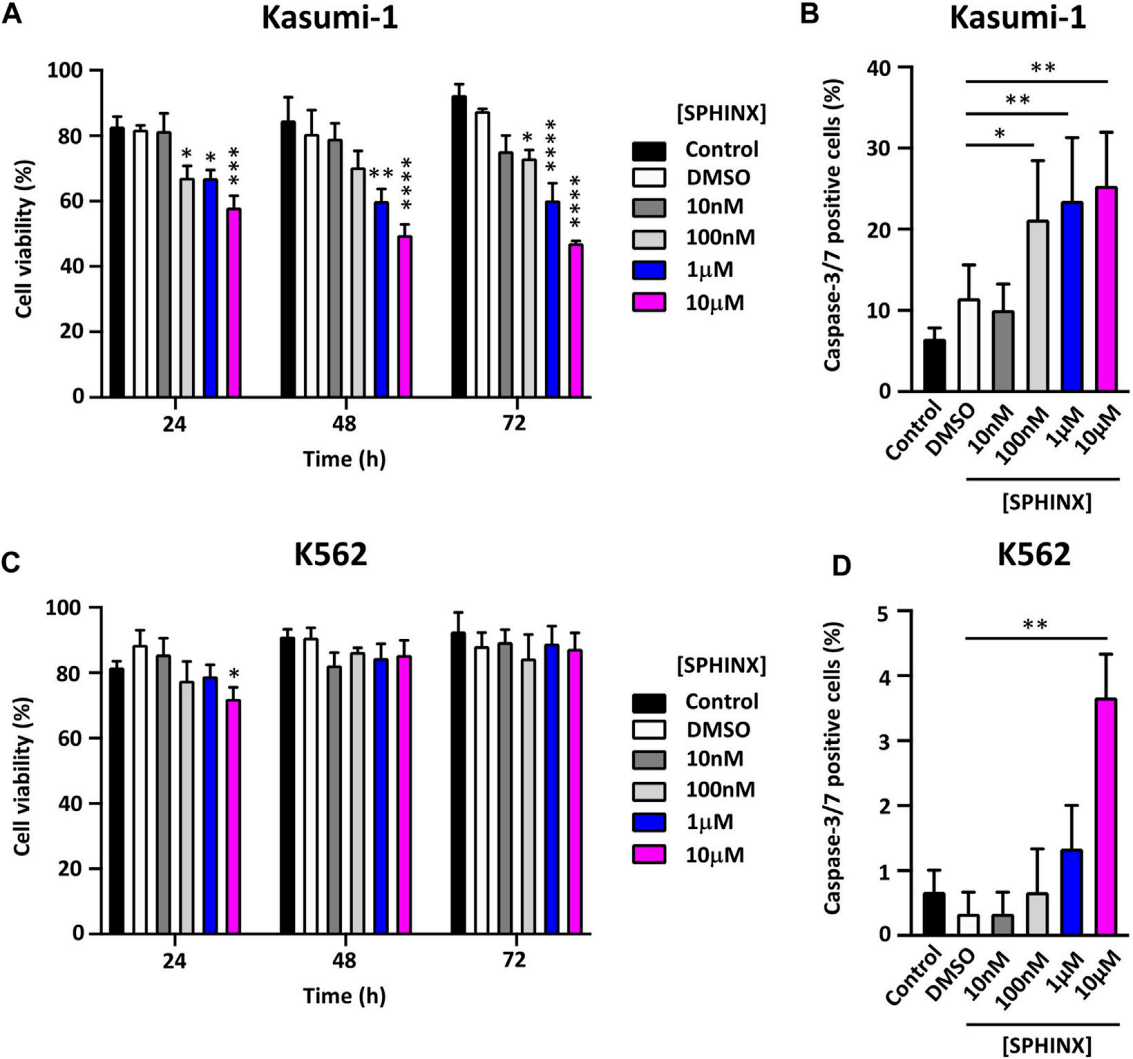
**(A)** Effect of SPHINX inhibition on cell viability (24 h *p ≤ 0.05, ***p = 0.0001; 48 h **p = 0.0010, ****p < 0.0001 and 72 h *p = 0.029, ****p < 0.0001) in Kasumi-1 cells. **(B)** Corresponding increase (*p = 0.01; **p < 0.001) in caspase-3/7 positive cells at 72 h post-treatment. Cell viability **(C,D)** caspase-3/7 positive cells, K562 cells. Two-way ANOVA (*n* = 3).

Having confirmed the effect of SPHINX on SR protein phosphorylation in Kasumi-1 cells, the effect of a range of SPHINX concentrations on cell viability was next examined. Concentrations ranged from 10 nM to 10 μM, and cell viability was assessed at 24, 48, and 72 h following a single dose of SPHINX (Figures 2A, C). In Kasumi-1 cells, 100 nM SPHINX already resulted in significant reductions in cell viability, with further dose-dependent effects on cell viability observed at 1 and 10 μM for 48 and 72 h (Figure 2A). However, K562 cells appeared less sensitive to SPHINX, with only a modest reduction in viability at 24 h with 10 μM dosing (Figure 2C). To complement these viability analyses, cell death was measured by activated caspase-3/7 staining following SPHINX treatment (Figures 2B, D). In Kasumi-1 cells, 100 nM SPHINX increased apoptosis significantly, with dose-dependent effects again observed 72 h following 1 and 10 μM treatments (Figure 2B). A significant increase in apoptotic cells was also observed in K562 cells, but only at the higher concentration of 10 μM SPHINX (Figure 2D). Together these results suggest that Kasumi-1 (AML) cells may be more sensitive to SRPK1 inhibition by SPHINX than K562 (CML) cells.

### Effect of siRNA-Mediated SRPK1 Knockdown on Cell Viability

**FIGURE 3:**
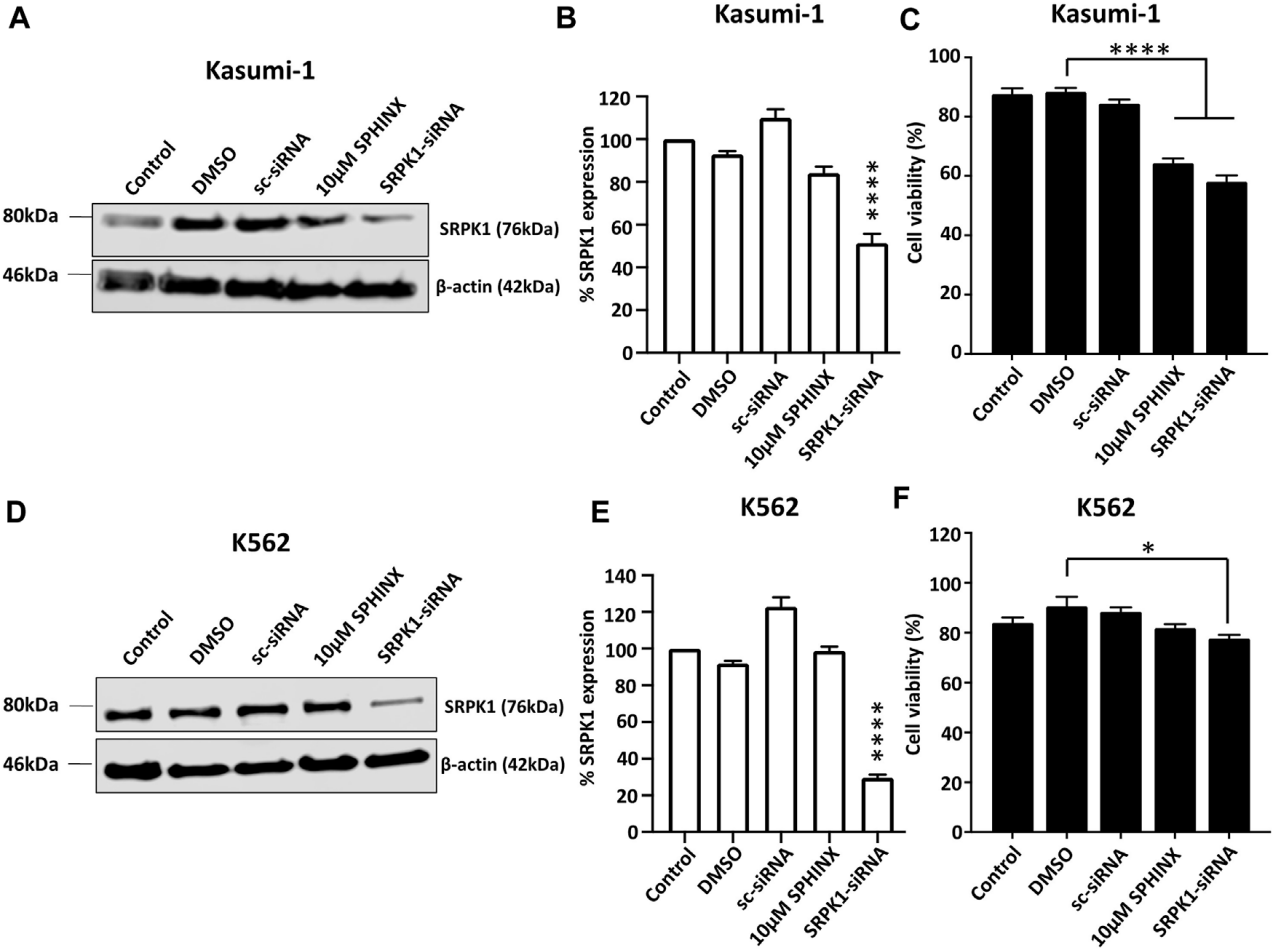
Effects of SPHINX inhibition and siRNA knockdown of SRPK1 in Kasumi-1 and K562 cells. **(A,D)** Representative western blots for Kasumi-1 and K562 cells respectively. Verification of knockdown by western blotting for SRPK1 48 h after siRNA transfection in Kasumi-1 **(B)** and K562 cells **(E)**. Statistical comparisons here are versus control. Corresponding cell viability changes shown in **(C,F)** for Kasumi-1 and K562 cells respectively with statistically significant comparisons indicated. One-way ANOVA (*n* = 3). **** = *p* < 0.0001.

To further examine the sensitivity of the cell lines to the loss of SRPK1 activity, the effect on cell viability of SPHINX treatment was compared with that of siRNA-mediated SRPK1 knockdown. Significant SRPK1 knockdown in both Kasumi-1 and K562 cells was first demonstrated by western blotting (SRPK1-siRNA lanes, Figures 3A, B, D, E). In line with our initial findings using SPHINX treatment, SRPK1 knockdown in Kasumi-1 cells significantly reduced cell viability (Figure 3C). Interestingly, in contrast to SPHINX treatment, significant effects on K562 cell viability following siRNA-mediated SRPK1 knockdown were also observed (Figure 3F). Furthermore, in Kasumi-1 cells, a decrease in pSRSF2, pSRSF4 and pSRSF5 following siRNA-mediated SRPK1 knockdown were also observed (Supplementary Figure S3A). Conversely, there appeared to be little change in pSRSF levels in the K562 cells following knockdown (Supplementary Figure S3B).

### Effect of Combining SPHINX With Azacitidine or Imatinib on Cell Viability and Apoptosis

**FIGURE 4:**
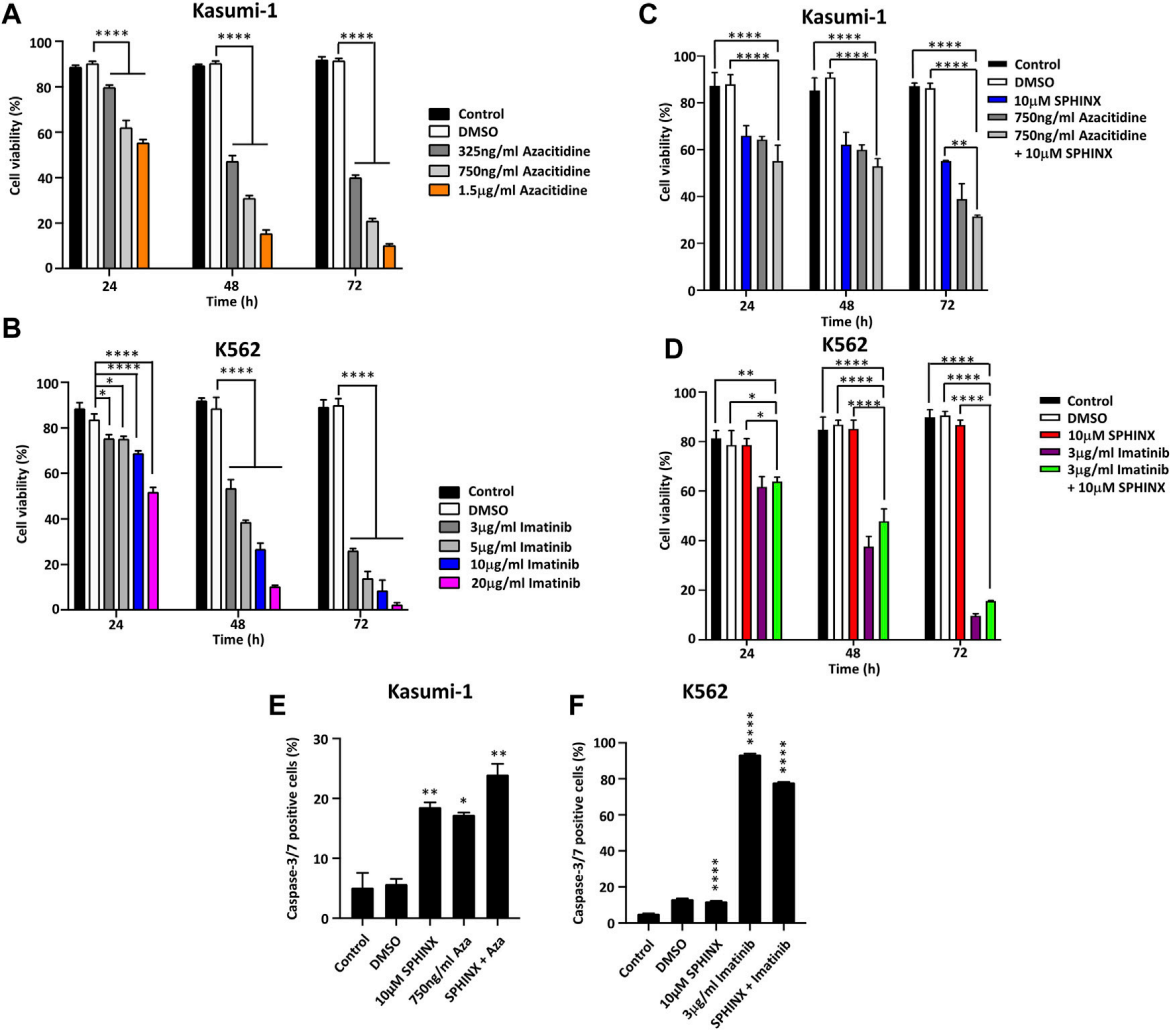
**(A)** Effects of azacitidine and **(B)** imatinib on cell viability in Kasumi-1 and K562 cells respectively, at 24, 48 and 72 h. The effect on cell viability of combining 10 µM SPHINX with 750 ng/ml azacitidine in Kasumi-1 cells **(C)** and 3 μg/ml imatinib in K562 cells **(D)** at 24, 48 and 72 h. Levels of caspase-3/7 staining 72 h after combined SPHINX and drug treatment in Kasumi-1 cells **(E)** and K562 cells **(F)**. One-way ANOVA (*n* = 3). *p < 0.05, **p < 0.01, ***p < 0.001 and ****p < 0.0001.

Previous reports have noted the sensitivity of Kasumi-1 cells to the DNA methyltransferase inhibitor azacitidine (30, 31) and that the tyrosine kinase inhibitor imatinib is effective against BCR-ABL positive K562 cells (32, 33). Kasumi-1 cells were exposed to 325 ng/ml–1.5 μg/ml azacitidine and K562 cells to 3–20 μg/ml imatinib for 24, 48 and 72 h followed by assessment of cell viability. As expected, substantial decreases in cell viability for both cell lines at these drug doses and for all timepoints were observed (Figures 4A, B). Next, these treatments were combined with 10 μM SPHINX to see if SPHINX potentiated the effects of azacitidine and imatinib (Figures 4C, D). As previously shown, Kasumi-1 cells exhibited reduced viability when exposed to 10 μM SPHINX at 24–72 h (Figure 4C). When combining 750 ng/ml azacitidine with 10 μM SPHINX in the Kasumi-1 cells, a further decrease in cell viability versus 10 μM SPHINX or 750 ng/ml azacitidine alone was observed (Figure 4C). Consistent with earlier findings, the K562 cells did not appear to be sensitive to SPHINX alone and combining 3 μg/ml imatinib with 10 μM SPHINX had no apparent additional effect on cell viability (Figure 4D). Finally, the effect of combining SPHINX with azacitidine and imatinib on apoptosis was assessed (Figures 4E, F). Consistent with our previous findings, SPHINX alone significantly increased apoptosis in Kasumi-1 cells; this was also observed with azacitidine alone (Figure 4E). 10μM SPHINX in combination with 750 ng/ml azacitidine further increased apoptosis in the Kasumi-1 cells. For the K562 cells, 3 μg/ml imatinib monotherapy substantially increased the number of apoptotic cells, with no additive effect on apoptosis when combined with 10 μM SPHINX (Figure 4F).

## Discussion

In haematological malignancies, mutations have been reported in genes that encode >30 splicing factors with proven or emerging roles in pre-mRNA splicing and its regulation (34). Growing evidence clearly points to the splice factor kinases playing key roles in cancer biology thereby presenting novel and attractive targets for the development of new therapies, potentially combining inhibitors of these splice factor kinases with standard-of-care drug treatments. The splice factor kinases that show promise as targets include the SR protein kinases (SRPKs) and CDC2-like protein kinases (CLKs) (12). Targeting CLKs with the small molecule inhibitor SM09419 in *TP53* mutant AML models results in downregulation of the Wnt signalling pathway and potent anti-tumour effects (35). It therefore seems likely that targeting several splice factor kinases, perhaps with a “cocktail” of inhibitors, could prove to be beneficial in the treatment of AML and other leukaemias, particularly those in which the expression of oncogenic splice isoforms is especially dependent on the activity of splice factor kinases.

Despite the evident promise of targeting splice factor kinases, Wang et al. emphasize an important caveat that applies to many other cancer-associated proteins—namely, that SRPK1 can potentially act as both an oncogene or tumour suppressor depending on the context. This functional complexity arises through its ability to modulate the activation state of Akt through interaction with the Akt phosphatase PHLPP1 (36). As such, under- or over-expression of SRPK1, can lead to constitutive Akt activation, offering a potential explanation for observations that SRPK1 levels can be downregulated or upregulated in different cancers. Understanding the mechanisms that underpin both activities in human tumours will be important for effective targeting of SRPK1 by cancer therapeutics. To add further complexity, there is another member of the SRPK family in humans, SRPK2, first cloned in 1998 (34). SRPK2 is very similar to SRPK1 in terms of primary sequence, kinase activity and substrate specificity (37). SRPK2 is less well studied, but there is evidence that it is also involved in cancer including leukaemia. In leukaemic cells SRPK2 binds and phosphorylates acinus, an SR protein splice factor, resulting in the upregulation of cyclin A1 expression and increased cell proliferation (38). Therefore, dual therapeutic targeting of SRPK1 and SRPK2 may be necessary to avoid any functional redundancy masking drug effects. To this end, Hatcher and colleagues recently describe SRPKIN-1, a covalent and potent inhibitor of both SRPK1 and SRPK2. SRPKIN-1 efficiently promotes the upregulation of anti-angiogenic VEGFA165b and blocks neovascularisation in a mouse retinal model (39).

In the present study we have focused on evaluating the effect of the SRPK1 inhibitor SPHINX (26) on two well-studied and established cell line models of leukaemia; Kasumi-1, representing acute myeloid leukaemia (AML), and K562 representing chronic myeloid leukaemia (CML). We have observed differential responses in the two cell lines to SRPK1 inhibition by SPHINX. The AML cell line Kasumi-1 appears to be more sensitive to either SRPK1 inhibition by SPHINX or SRPK1 knockdown compared to the CML cell line K562. We also observe that combining SPHINX with established drugs does not augment effects in the case of K562 cells (SPHINX plus imatinib) but appears to enhance the potency of azacitidine in Kasumi-1 cells. Identifying this mechanism of action will form the basis of future studies. Furthermore, our interesting observation that relative levels of SRPK1 and SRPK2 are anti-correlated with SPHINX sensitivity, i.e., low relative levels of SRPK1/2 equate to SPHINX sensitivity in Kasumi-1, warrants further investigation. Whilst we demonstrate that SPHINX is clearly acting through modulation of SRPK1 and interacting SRSFs, SRPK1 levels themselves may not serve as a robust biomarker of response to SRPK1. Related to our findings in this context, a recent large-scale study examined the sensitivity of hundreds of cancer cell lines to hundreds of drugs, which was correlated with expression of drug targets within the cells. Here, Roy et al. described inverse correlations between target expression and drug sensitivity for 8% of targets, suggesting drug efficacy may not only be determined by expression levels of the drug target, but may also depend on other factors such as genetic background and other molecules that could affect drug-target interactions, including the expression of other gene family members or interacting proteins (40).

There is evidence that mutations that affect the pre-mRNA splicing machinery are especially prominent in AML, and that they are associated with drug resistance through altered splicing of cancer-associated genes, including genes associated with apoptosis (41). We observe a prominent increase in apoptosis in SPHINX-treated AML and CML cells, suggesting that SRPK1 plays a central role in the regulation of alternative splicing, presumably by favouring the expression of anti-apoptotic splice isoforms. This has been observed in breast cancer cells, in which elevated SRPK1 reduces apoptosis through RBM4-regulated alternative splicing (14). SRPK1 activity also appears to counteract apoptosis in colon cancer cells (42) and might therefore be a general mechanism through which SRPK1 is involved in cancer, including AML. The avoidance of apoptosis is a key cancer hallmark, often associated with resistance to chemotherapy. As such, there is significant interest in developing drugs that promote apoptosis in AML, including for example, drugs that target apoptosis regulators such as BCL2 and MCL1 (43, 44).

In summary, we suggest that in the context of AML, and potentially in other types of leukaemia, there may be therapeutic potential in targeting SRPK1 and other splice factor kinases. We envisage that in the future splice factor kinase inhibitors could be used in combination with both well-established and novel drugs for the eventual clinical management of AML treatment.

## Conclusion

The dysregulation of alternative splicing is a prominent feature of cancer, presenting opportunities for the exploration of novel drug targets. Alternative splicing is regulated by splice factors whose activity is modulated by splice factor kinases. Elevated splice factor kinase activity is observed in several cancer types, including breast, lung and haematological malignancies. The purpose of this study was to examine the effect of the SRPK1 splice factor kinase inhibitor SPHINX on proliferation and apoptosis in two leukaemic cell line models, Kasumi-1 and K562, alone and in combination with the established drugs azacitidine and imatinib.

SPHINX inhibition of SRPK1 reduced the proliferation of and significantly increased rates of apoptosis in the acute myeloid leukaemia cell line Kasumi-1. Kasumi-1 cells are more sensitive to SPHINX than the chronic myeloid leukaemia cell line K562. Combining SPHINX with the clinically-used drug azacitidine potentiated these effects in Kasumi-1 cells. These results highlight the need to continue exploring targeting splice factor kinases such as SRPK1 in leukaemias, particularly in combination with standard-of-care therapies, as well as in other cancers where splice factor kinase activity is elevated.

## Summary Table

### What is Known About This Subject

• Aberrant alternative splicing is implicated in many cancers and plays a prominent role in the development and progression of different types of leukaemia.
• The activity of splice factors is enhanced through their phosphorylation by splice factor kinases that include the SRPKs and CLKs.
• SRPK1 inhibition by SPHINX, a potent and specific inhibitor of SRPK1, modifies the alternative splicing of key cancer associated genes such as *VEGFA.*

### What This Paper Adds

• SPHINX reduces cell proliferation and increases apoptosis in leukaemic cell lines.
• The effectiveness of SPHINX is cell-line dependent and therefore some types of leukaemia such as AML may be more sensitive to splice factor kinase inhibition.
• Combining an effective splice factor kinase inhibitor (e.g., SPHINX) with established chemotherapeutic drugs such as azacitidine could potentially augment their clinical effectiveness.

## Summary Sentence

This work further underlines the importance of targeting the machinery of alternative splicing in leukaemias. There is a need to develop potent and specific splice factor kinase inhibitors.

## Data Availability

The original contributions presented in the study are included in the article/Supplementary Material, further inquiries can be directed to the corresponding authors.

## Supplementary Material

Supplementary Figure S1
**(A)** Mutation analyses of SRPKs and SRSFs in Kasumi-1 and K562 cells. **(B)** Relative expression (Z-score) of SRPK1 (left) and SRPK2 (right) in Kasumi-1 and K562 cell lines. **(C)** Relative SRPK1 (top) and SRPK2 (bottom) expression (Z-score) in a panel of AML cell lines. Arrows denote Kasumi-1 values. **(D)** Relative SRPK1 (top) and SRPK2 (bottom) expression (Z-score) in a panel of CML cell lines. Arrows denote K562 values. Mutation and protein expression data was downloaded from the Cell Model Passports database (https://cellmodelpassports.sanger.ac.uk/).

Supplementary Figure S2 Representative western blot image showing levels of SR protein phosphorylation levels in K562 cells treated with SPHINX for 24 h at indicated doses, and time-matched β-actin as loading control.

Supplementary Figure S3 Effects of SPHINX inhibition and siRNA knockdown of SRPK1 in Kasumi-1 and K562 cells on pSRSF protein levels. **(A,B)** Representative western blots for Kasumi-1 and K562 cells respectively, with β-actin as loading control.

## References

[1] Pan Q Shai O Lee LJ Frey BJ Blencowe BJ. Deep Surveying of Alternative Splicing Complexity in the Human Transcriptome by High-Throughput Sequencing. Nat Genet (2008) 40(8):1413–5. 10.1038/ng.259 18978789

[2] Wang ET Sandberg R Luo S Khrebtukova I Zhang L Mayr C Alternative Isoform Regulation in Human Tissue Transcriptomes. Nature (2008) 456(7221):470–6. 10.1038/nature07509 18978772 PMC2593745

[3] Park E Pan Z Zhang Z Lin L Xing Y. The Expanding Landscape of Alternative Splicing Variation in Human Populations. Am J Hum Gen (2018) 102(1):11–26. 10.1016/j.ajhg.2017.11.002 PMC5777382 29304370

[4] Cherry S Lynch KW. Alternative Splicing and Cancer: Insights, Opportunities, and Challenges from an Expanding View of the Transcriptome. Genes Dev (2020) 34(15-160):1005–16. 10.1101/gad.338962.120 32747477 PMC7397854

[5] Das S Krainer AR. Emerging Functions of SRSF1, Splicing Factor and Oncoprotein, in RNA Metabolism and Cancer. Mol Cancer Res (2014) 12(9):1195–204. 10.1158/1541-7786.MCR-14-0131 24807918 PMC4163531

[6] Aubol BE Plocinik RM Keshwani MM mcgione ML Hagopian JC Ghosh G N-terminus of the Protein Kinase CLK1 Induces SR Protein Hyper-Phosphorylation. Biochem J (2014) 462(1):143–52. 10.1042/BJ20140494 24869919 PMC5056641

[7] Ghosh G Adams JA. Phosphorylation Mechanism and Structure of Serine-Arginine Protein Kinases. FEBS J (2011) 278(4):587–97. 10.1111/j.1742-4658.2010.07992.x 21205204 PMC3079193

[8] Kuroyanagi M Kimura T Wada K Hisamoto N Matsumoto K Hagiwara M. SPK-1, a *C.Elegans* SR Protein Kinase Homologue, Is Essential for Embryogenesis and Required for Germline Development. Mech Dev (2000) 99(1-2):51–64. 10.1016/s0925-4773(00)00477-9 11091073

[9] Galvin BD Dennig DP Horvitz HR. SPK-1, an SR Protein Kinase, Inhibits Programmed Cell Death in *Caenorhabditis elegans* . Proc Natl Acad Sci USA (2011) 108(5):1998–2003. 10.1073/pnas.1018805108 21245325 PMC3033281

[10] Gou LT Lim DH Ma W Aubol BE Hao Y Wang X Initiation of Parental Genome Reprogramming in Fertilized Oocyte by Splicing Kinase SRPK1-Catalyzed Protamine Phosphorylation. Cell (2020) 180(6):1212–27.e14. 10.1016/j.cell.2020.02.020 32169215 PMC7190278

[11] Bullock N Oltean S. The Many Faces of SRPK1. J Pathol (2017) 241(4):437–40. 10.1002/path.4846 27859253 PMC5324686

[12] Corkery DP Holly AC Lahsaee S Dellaire G. Connecting the Speckles: Splicing Kinases and Their Role in Tumorigenesis and Treatment Response. Nucleus (2015) 6(4):279–88. 10.1080/19491034.2015.1062194 26098145 PMC4615201

[13] Wang C Zhou Z Subhramanyam CS Cao Q Heng ZSL Liu W SRPK1 Acetylation Modulates Alternative Splicing to Regulate Cisplatin Resistance in Breast Cancer Cells. Commun Biol (2020) 3(1):268. 10.1038/s42003-020-0983-4 32461560 PMC7253463

[14] Lin JC Lin CY Tarn WY Li FW. Elevated SRPK1 Lessens Apoptosis in Breast Cancer Cells through RBM4-Regulated Splicing Events. RNA (2014) 20(10):1621–31. 10.1261/rna.045583.114 25140042 PMC4174443

[15] van Roosmalen W Dévédec SE Golani O Smid M Pulyakhina I Timmermans AM Tumor Cell Migration Screen Identifies SRPK1 as Breast Cancer Metastasis Determinant. J Clin Invest (2015) 125(4):1648–64. 10.1172/JCI74440 25774502 PMC4396474

[16] Malvi P Janostiak R Chava S Manrai P Yoon E Singh K LIMK2 Promotes the Metastatic Progression of Triple-Negative Breast Cancer by Activating SRPK1. Oncogenesis (2020) 9(8):77. 10.1038/s41389-020-00263-1 32859889 PMC7455732

[17] Nikas IP Themistocleous SC Paschou SA Tsamis KI Ryu HS. Serine-Arginine Protein Kinase 1 (SRPK1) as a Prognostic Factor and Potential Therapeutic Target in Cancer: Current Evidence and Future Perspectives. Cells (2019) 9(1):19. 10.3390/cells9010019 31861708 PMC7017105

[18] Yi N Xiao M Jiang F Liu Z Ni W Ni R SRPK1 Is a Poor Prognostic Indicator and a Novel Potential Therapeutic Target for Human Colorectal Cancer. Onco Targets Ther (2018) 11:5359–70. 10.2147/OTT.S172541 30214242 PMC6128266

[19] Gonçalves V Henriques AFA Matos P Jordan P. Ibuprofen Disrupts a WNK1/GSK3β/SRPK1 Protein Complex Required for Expression of Tumor-Related Splicing Variant RAC1B in Colorectal Cells. Oncotarget (2020) 11(47):4421–37. 10.18632/oncotarget.27816 33315986 PMC7720772

[20] Liu H Gong Z Li K Zhang Q Xu Z Xu Y. SRPK1/2 and PP1α Exert Opposite Functions by Modulating SRSF1-Guided *MKNK2* Alternative Splicing in Colon Adenocarcinoma. J Exp Clin Cancer Res (2021) 40(1):75. 10.1186/s13046-021-01877-y 33602301 PMC7893936

[21] Tzelepis K Koike-Yusa H de Braekeleer E Li Y Metzakopia E Dovey OM A CRISPR Dropout Screen Identifies Genetic Vulnerabilities and Therapeutic Targets in Acute Myeloid Leukemia. Cell Rep (2016) 17(4):1193–205. 10.1016/j.celrep.2016.09.079 27760321 PMC5081405

[22] Tzelepis K de Braekeleer E Aspris D Barbieri I Vijayabaskar MS Liu WH SRPK1 Maintains Acute Myeloid Leukemia through Effects on Isoform Usage of Epigenetic Regulators Including BRD4. Nature (2018) 9(1):5378. 10.1038/s41467-018-07620-0 PMC6300607 30568163

[23] Nowak DG Amin EM Rennel ES Hoareu-Aveilla C Gammons M Damodaran G Regulation of Vascular Endothelial Growth Factor (VEGF) Splicing from Pro-angiogenic to Anti-angiogenic Isoforms: A Novel Therapeutic Strategy for Angiogenesis. J Biol Chem (2010) 285(8):5532–40. 10.1074/jbc.M109.074930 19906640 PMC2820781

[24] Amin EM Oltean S Hua J Gammons MV Hamdollah-Zadeh M Welsh GI WT1 Mutants Reveal SRPK1 to Be a Downstream Angiogenesis Target by Altering VEGF Splicing. Cancer Cell (2011) 20(6):768–80. 10.1016/j.ccr.2011.10.016 22172722 PMC3574979

[25] Mavrou A Brakspear K Hamdollah-Zadeh M Damodaran G Babaei-Jadidi R Oxley J Serine-arginine Protein Kinase 1 (SRPK1) Inhibition as a Potential Novel Targeted Therapeutic Strategy in Prostate Cancer. Oncogene (2015) 34(33):4311–9. 10.1038/onc.2014.360 25381816 PMC4351909

[26] Batson J Toop HD Redondo C Babaei-Jadidi R Chaikuad A Wearmouth SF Development of Potent, Selective SRPK1 Inhibitors as Potential Topical Therapeutics for Neovascular Eye Disease. ACS Chem Biol (2017) 12(3):825–32. 10.1021/acschembio.6b01048 28135068

[27] Gammons MV Fedorov O Ivison D Du C Clark T Hopkins C Topical Antiangiogenic SRPK1 Inhibitors Reduce Choroidal Neovascularization in Rodent Models of Exudative AMD. Invest Ophthalmol Vis Sci (2013) 54(9):6052–62. 10.1167/iovs.13-12422 23887803 PMC3771558

[28] Van der Meer D Barthorpe S Yang W Lightfoot H Hall C Gilbert J Cell Model Passports – a Hub for Clinical, Genetic and Functional Datasets of Preclinical Cancer Models. Nuc Acids Res (2019) 47(D1):D923–D929. 10.1093/nar/gky872 PMC6324059 30260411

[29] Belali T Wodi C Clark B Cheung MK Craig TJ Wheway G WT1 Activates Transcription of the Splice Factor Kinase *SRPK1* Gene in PC3 and K562 Cancer Cells in the Absence if Corepressor BASP1. Biochim Biophys Acta Gene Regul Mech (2020) 1863(12):194642. 10.1016/j.bbagrm.2020.194642 33017668

[30] Flotho C Claus R Batz C Schneider M Sandrock I Ihde S The DNA Methyltransferase Inhibitors Azacitidine, Decitabine and Zebularine Exert Differential Effects on Cancer Gene Expression in Acute Myeloid Leukemia Cells. Leukemia (2009) 23(6):1019–28. 10.1038/leu.2008.397 19194470

[31] Derissen EJB Beijnen JH Schellens JHM. Concise Drug Review: Azacitidine and Decitabine. New Drug Dev Clin Pharm (2013) 18(5):619–24. 10.1634/theoncologist.2012-0465 PMC3662854 23671007

[32] Azevedo LD Bastos MM Vasconcelos F Hoelz LVB Junior FPS Dantas RF Imatinib Derivatives as Inhibitors of K562 Cells in Chronic Myeloid Leukemia. Med Chem Res (2017) 26:2929–41. 10.1007/s00044-017-1993-8

[33] Cortes JE Egorin MJ Guilhot F Molimard M Mahon FX. Pharmacokinetic/Pharmacodynamic Correlation and Blood-Level Testing in Imatinib Therapy for Chronic Myeloid Leukemia. Leukemia (2009) 23(9):1537–44. 10.1038/leu.2009.88 19404318

[34] Saez B Walter MJ Graubert TA. Splicing Factor Gene Mutations in Hematologic Malignancies. Blood (2017) 129(10):1260–9. 10.1182/blood-2016-10-692400 27940478 PMC5345729

[35] Chung H Creger E Sitts L Chiu K Mak CC Sunil KC SM09419, a Novel, Small-Molecule CDC-like Kinase (CLK) Inhibitor, Demonstrates Strong Inhibition of the Wnt Signaling Pathway and Antitumor Effects in Tumor Protein P53 (TP53)-Mutant Acute Myeloid Leukemia Models. Blood (2019) 134:3913. 10.1182/blood-2019-131209

[36] Wang P Zhou Z Hu A Ponte de Albuquerque C Zhou Y Hog L Both Decreased and Increased SRPK1 Levels Promote Cancer by Interfering with PHLPP-Mediated Dephosphorylation of Akt. Mol Cel (2014) 54(3):378–91. 10.1016/j.molcel.2014.03.007 PMC4019712 24703948

[37] Wang HY Lin W Dyck JA Yeakley JM Songyang Z Cantley LC SRPK2: a Differentially Expressed SR Protein-specific Kinase Involved in Mediating the Interaction and Localization of Pre-mRNA Splicing Factors in Mammalian Cells. J Cel Biol (1998) 140(4):737–50. 10.1083/jcb.140.4.737 PMC2141757 9472028

[38] Jang SW Yang S Ehlén A Dong S Khoury H Chen J Serine/arginine Protein-specific Kinase 2 Promotes Leukemia Cell Proliferation by Phosphorylating Acinus and Regulating Cyclin A1. Cancer Res (2008) 68(12):4559–70. 10.1158/0008-5472.CAN-08-0021 18559500 PMC2805021

[39] Hatcher JM Wu G Zeng C Zhu J Meng F Patel S SRPKIN-1: A Covalent SRPK1/2 Inhibitor that Potently Converts VEGF from Pro-angiogenic to Anti-angiogenic Isoform. Cell Chem Biol (2018) 25(4):460–70.e6. 10.1016/j.chembiol.2018.01.013 29478907 PMC5973797

[40] Winteringham LN Lassmann T Forrest ARR. Expression Levels of Therapeutic Targets as Indicators of Sensitivity to Targeted Therapeutics. Mol Cancer Ther (2019) 18(12):2480–9. 10.1158/1535-7163.MCT-19-0273 31467181

[41] de Necochea-Campion R Shouse GP Zhou Q Mirshahidi S Chen CS. Aberrant Splicing and Drug Resistance in AML. J Hematol Oncol (2016) 9(1):85. 10.1186/s13045-016-0315-9 27613060 PMC5018179

[42] Huang JQ Li HF Zhu J Song JW Zhang XB Gong P SRPK1/AKT axis Promotes Oxaliplatin-Induced Anti-apoptosis via NF-Κb Activation in colon Cancer. J Transl Med (2021) 19(1):280. 10.1186/s12967-021-02954-8 34193174 PMC8243872

[43] Cassier PA Castets M Belhabri A Vey N. Targeting Apoptosis in Acute Myeloid Leukaemia. Br J Cancer (2017) 117(8):1089–98. 10.1038/bjc.2017.281 29017180 PMC5674101

[44] McBride A Houtmann S Wilde L Vigil C Eischen CM Kasner M The Role of Inhibition of Apoptosis in Acute Leukemias and Myelodysplastic Syndrome. Front Oncol (2019) 9:192. 10.3389/fonc.2019.00192 30972300 PMC6445951

