# Supplementary figures and images for: SPHINX-Based Combination Therapy as a Potential Novel Treatment Strategy for Acute Myeloid Leukaemia
Source: Br J Biomed Sci. 2023 Feb 21;80:11041. doi: 10.3389/bjbs.2023.11041 (PMC9988938; doi:10.3389/bjbs.2023.11041)

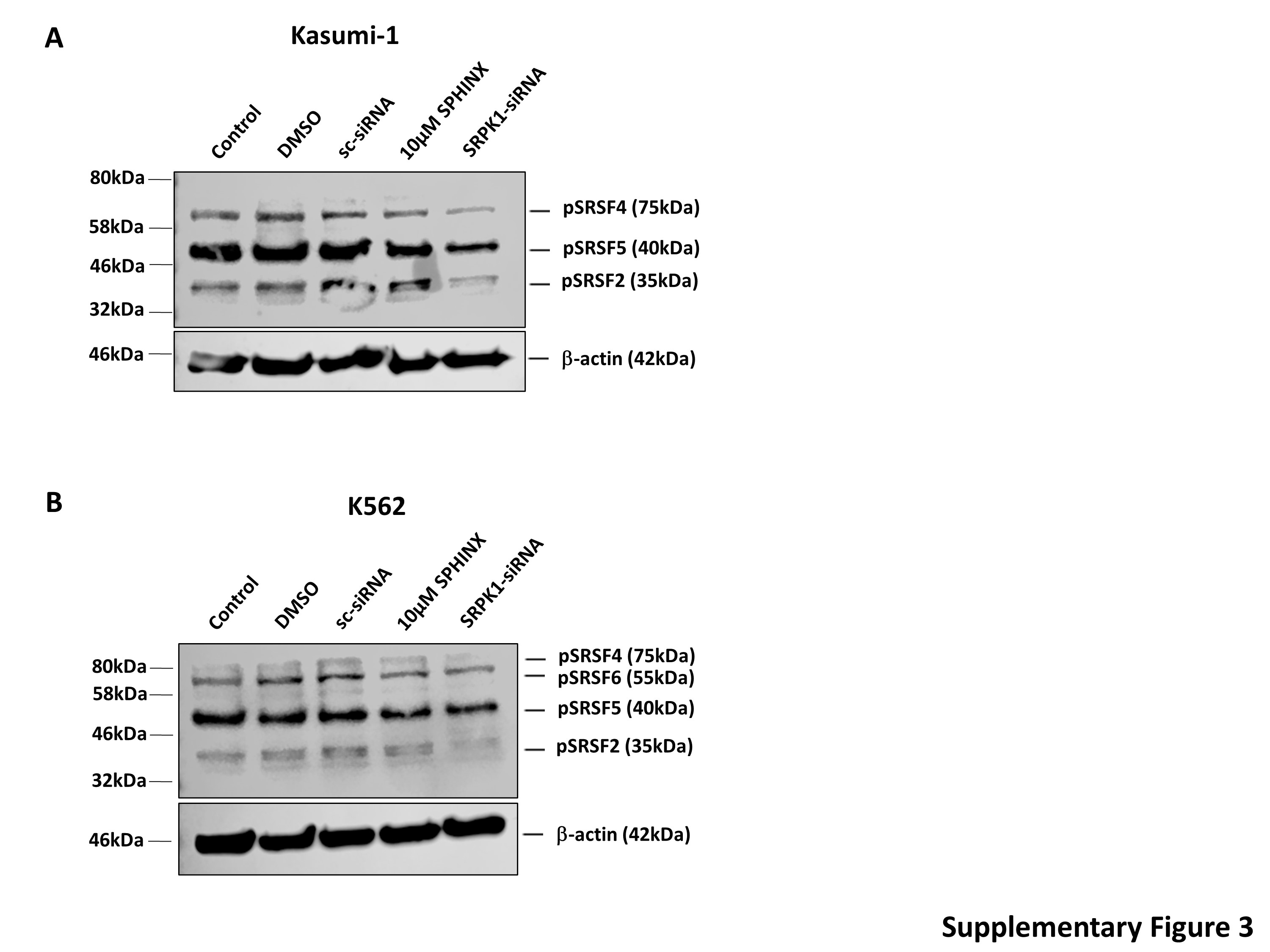

Supplement: Supplementary file 1 [file Image3.jpeg]

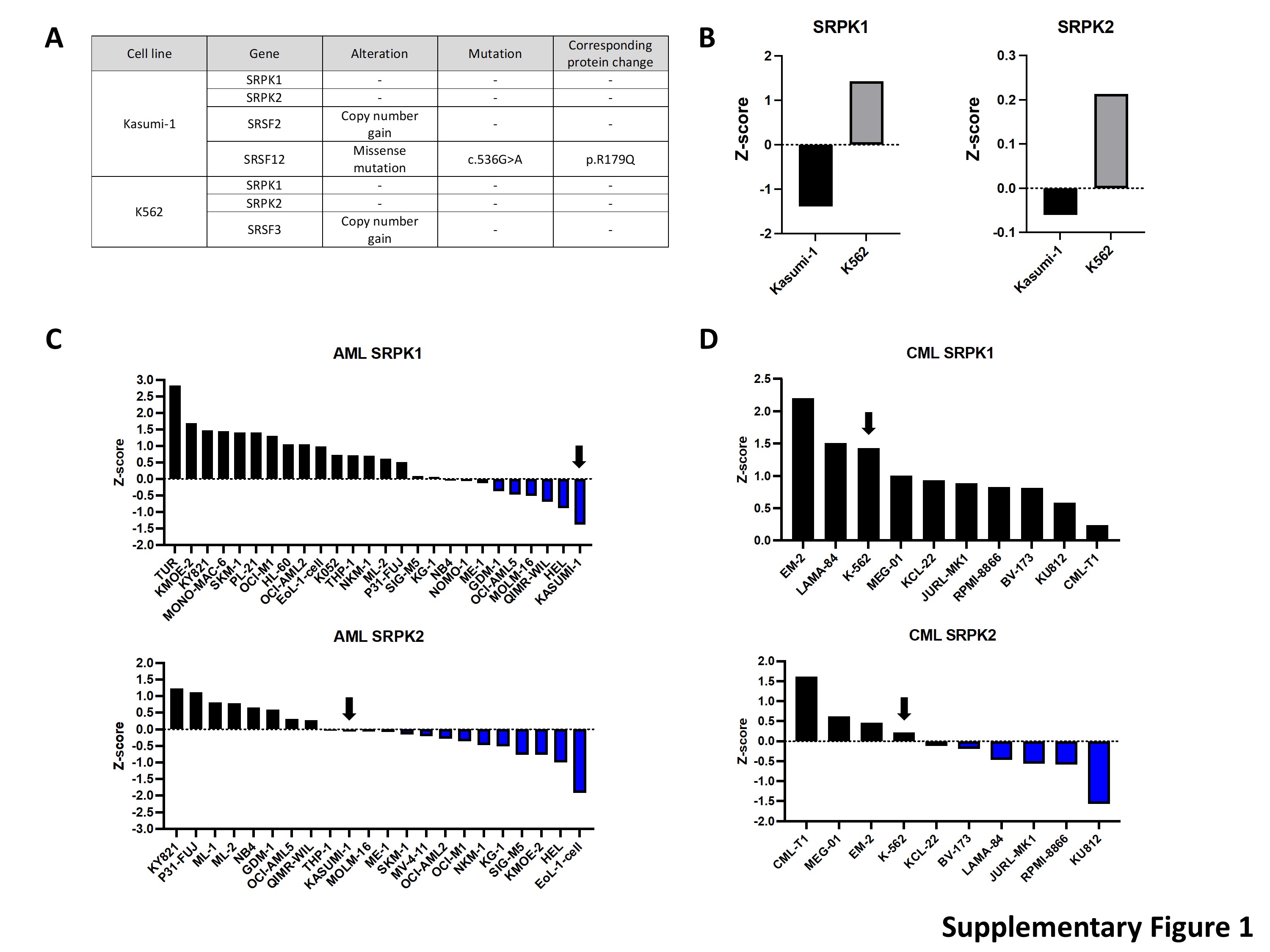

Supplement: Supplementary file 2 [file Image1.jpeg]

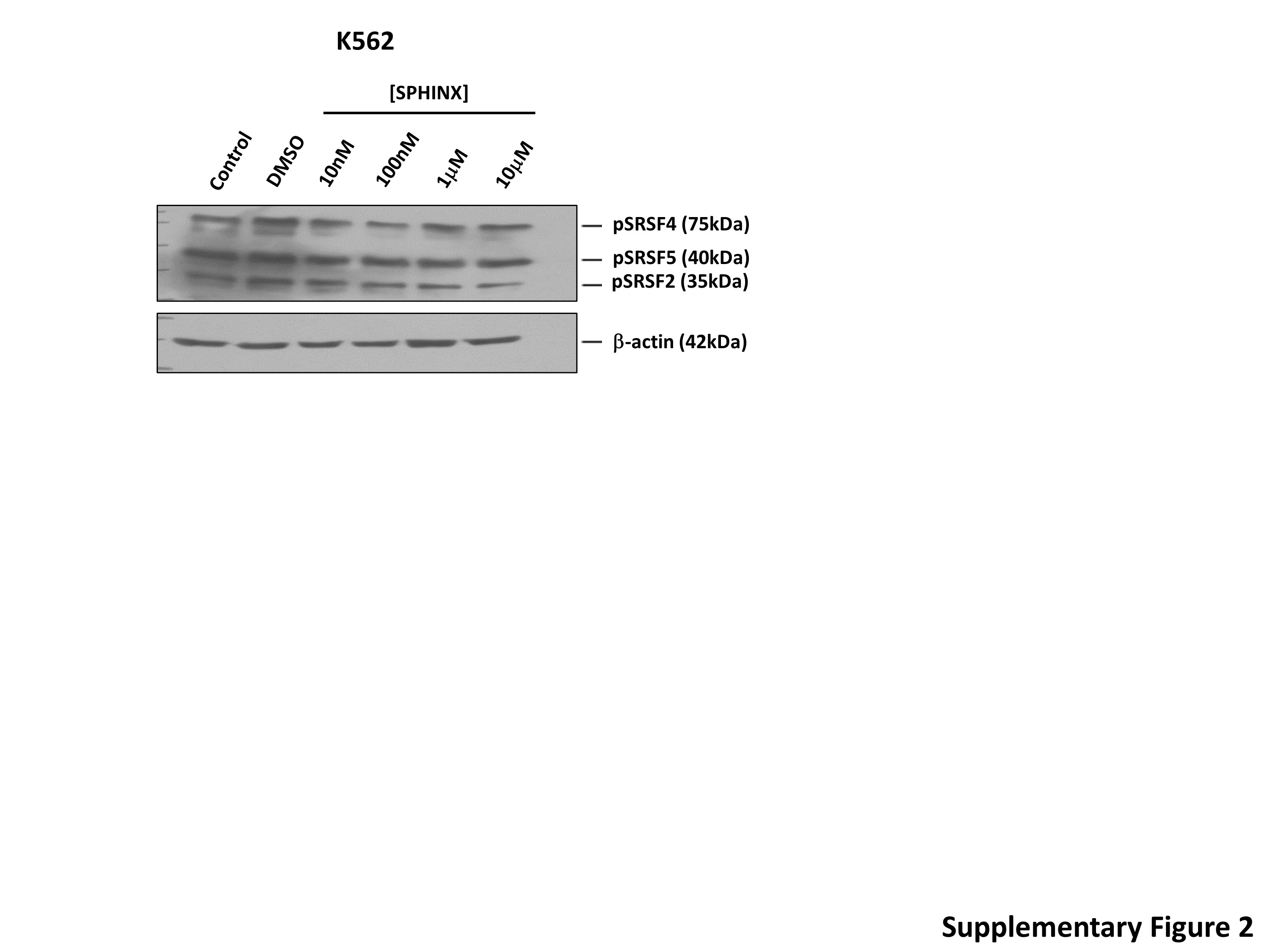

Supplement: Supplementary file 3 [file Image2.jpeg]
